# Reduced macular inner retinal thickness and microvascular density in the early stage of patients with dysthyroid optic neuropathy

**DOI:** 10.1186/s40662-020-00180-9

**Published:** 2020-03-10

**Authors:** Yufei Wu, Yunhai Tu, Chaoming Wu, Lulu Bao, Jianhua Wang, Fan Lu, Meixiao Shen, Qi Chen

**Affiliations:** 1 School of Ophthalmology and Optometry, Wenzhou Medical University, 270 Xueyuan Road, Wenzhou, 325027 Zhejiang China; 2 Department of Ophthalmology, Yinzhou Hospital Affiliated to Medical School of Ningbo University, Ningbo, Zhejiang China; 3 The Second Affiliated Hospital & Yuying Children’s Hospital of Wenzhou Medical University, Wenzhou, Zhejiang China; 4 Bascom Palmer Eye Institute, University of Miami Miller School of Medicine, Miami, FL USA; 5 Electrical and Computer Engineering, University of Miami, Miami, FL USA

**Keywords:** Retina, Microvasculature, Density, Optical coherence tomography angiography, Thyroid-associated ophthalmopathy

## Abstract

**Background:**

The goal was to investigate changes of the inner intra-retinal layer thicknesses and retinal capillary density (RCD) around the macula in thyroid-associated ophthalmopathy (TAO) patients with or without dysthyroid optic neuropathy (DON).

**Methods:**

Forty-four TAO patients including 23 non-DON and 21 DON patients, and 38 healthy participants were enrolled. Spectral domain optical coherence tomography equipped with Angiovue was used to obtain three-dimensional retinal thickness maps and microvascular images of the superficial and deep retinal capillary layers (SRCL and DRCL, respectively) around the macula. Quantitative analyses were performed using a custom automated algorithm.

**Results:**

The thicknesses of the nerve fiber layer, ganglion cell layer + inner plexiform layer, and ganglion cell complex (GCC) as well as the RCDs in the SRCL and DRCL in both TAO groups were significantly decreased compared to the controls. In addition, the RCDs in DRCL of the DON group were further decreased compared to the non-DON group. GCC thickness in both TAO groups was positively correlated with the RCDs of the SRCL in the total annular zone and in the temporal, inferior, and nasal sectors. The areas under the receiver operating characteristic curves for the GCC thickness combined with the RCD were generally larger than those of each single indicator.

**Conclusions:**

Thinned inner intra-retinal layers and decreased RCDs in the TAO patients without DON revealed that morphological changes might precede visual dysfunction. The composite index of the retinal structure and the microvascular density might be valuable in the diagnosing, monitoring, and intervention for early DON.

## Background

Graves’ disease is a progressive and organ specific autoimmune disease that typically affects the thyroid gland and the ocular orbit. Approximately 35–47% of patients with Graves’ disease have ophthalmic manifestations typically described as thyroid-associated ophthalmopathy (TAO) [1]. Although the manifestations are mild in the majority of TAO patients, 3–8% have a severe sight-threatening disease due to ocular neurological effects referred to as dysthyroid optic neuropathy (DON) [2–4]. Clinical diagnosis of DON is made based on decreased visual acuity, color vision loss, relative afferent pupillary defect when only one eye is involved, compatible visual field defects, and significant crowded orbital apex on CT scanning. However, it is sometimes difficult to identify the early involvement of the optic nerve and retinal tissue by these visual functional tests as the responses are all subject to change and the findings are not necessarily congruent [5]. Identifying early objective indicators in TAO even before the clinical diagnosis of DON will allow better monitoring, earlier intervention, and promote the development of new treatment methods.

The main pathogenic mechanisms of DON have been attributed to mechanical compression of the optic nerve and retinal tissue themselves [6] or abnormal blood supply [7] due to an increase in the volume of orbital contents [8], as well as the secondary elevation of intraocular pressure (IOP) [9, 10]. With the use of optical coherence tomography (OCT), thickness changes of the peripapillary retinal nerve fiber layer (P-RNFL) [11, 12] and retina around the macula [12, 13] were found in TAO patients and were correlated with the visual dysfunction in DON. Previous articles also reported retinal arterial stenosis, venous stasis, and increased resistance indexes in branches of central retinal artery, indicating the presence of hypoxia and ischemia in TAO patients [7, 12, 14]. Moreover, Sayin et al. [13] proposed that the abnormal blood supply might induce retinal structural changes before the mechanical compression developed.

Health of the macula is of great importance in maintaining normal visual functions as it comprises about 30% of all retinal ganglion cells and is responsible for over 50% of the input to the visual cortex. Spectral domain optical coherence tomography (SD-OCT) has sufficiently high resolution to enable detailed in vivo detection of intra-retinal structures that were previously available only by histopathology [15, 16]. OCT angiography (OCT-A) is a novel imaging modality that non-invasively and quickly obtains high resolution images of the retinal microvasculature, allowing in-depth visualization of the retinal microvascular network in the different retinal layers [17–19]. In this study, we investigated the structure of the inner intra-retinal layers and the microvascular network around the macula of healthy controls and of TAO patients with and without clinically diagnosed DON. We documented the changes in the diseased eyes and assessed the ability of the changes to indicate early retinal evidence of DON.

## Methods

### Subjects

This prospective study was performed at a single center and in accordance with the tenets of the Declaration of Helsinki. This study was approved by The Eye Hospital of Wenzhou Medical University ethics committee, which approved the screening, inspection, and patient data collection. All patients provided written informed consent prior to participating in the study.

All patients underwent a series of ocular examinations, including refraction and best corrected visual acuity (BCVA) test, slit-lamp biomicroscopy, axial length measurement, IOP measurement, exophthalmometry measurements, visual field test by a Humphrey Field Analyzer, color vision test, and ophthalmoscopy. The demographic information of all patients was collected, including age, sex as well as laboratory serum biochemical indicators, including free triiodothyronine (FT3), free thyroxine (FT4), and thyroid-stimulating hormone (TSH).

All the patients were derived from the Endocrinology Department of the Second Affiliated Hospital & Yuying Children’s Hospital of Wenzhou Medical University and were diagnosed as hyperthyroidism by an endocrine specialist (CW) according to the laboratory evidence. Then, they were taken to the Eye Hospital of Wenzhou Medical University for the further examination by an orbital disease ophthalmologist (YT). Those diagnosed as TAO were enrolled in the current study, according to the Bartley international diagnostic criteria [20]. The clinical activity scoring (CAS) system was used to establish the disease activity score in TAO patients [21]. In addition, TAO patients were further divided into DON and non-DON groups [22]. DON was diagnosed based on decreased BCVA (≤ 20/25), apparent visual field changes with a mean defect (MD) < − 1 dB, relative afferent pupillary defect when unilaterally affected, decreased color vision, abnormal optic nerve head, as well as evident apical crowding in orbital CT. TAO patients with normal BCVA (better than 20/25), visual field (MD ≥ − 1 dB), color vision, optic nerve head, and CT scans were assigned to the non-DON group. Exclusion criteria were as follows: refractive errors over + 2.00 diopters (D) or under − 3.00 D of spherical equivalent or − 1.50 D of astigmatism, significant media opacities, IOP > 21 mmHg or previous diagnosis of glaucoma, uveitis, retinal diseases or vasculitis, as well as autoimmune and systemic diseases like hypertension and retinopathy due to diabetes. Age- and sex-matched control subjects were recruited from workers at The Eye Hospital of Wenzhou Medical University and family members of patients at the same hospital. Control individuals underwent the same tests as those used to evaluate the TAO patients, except for laboratory serum biochemical indicators and visual field tests.

### OCT imaging procedure

**Fig. 1:**
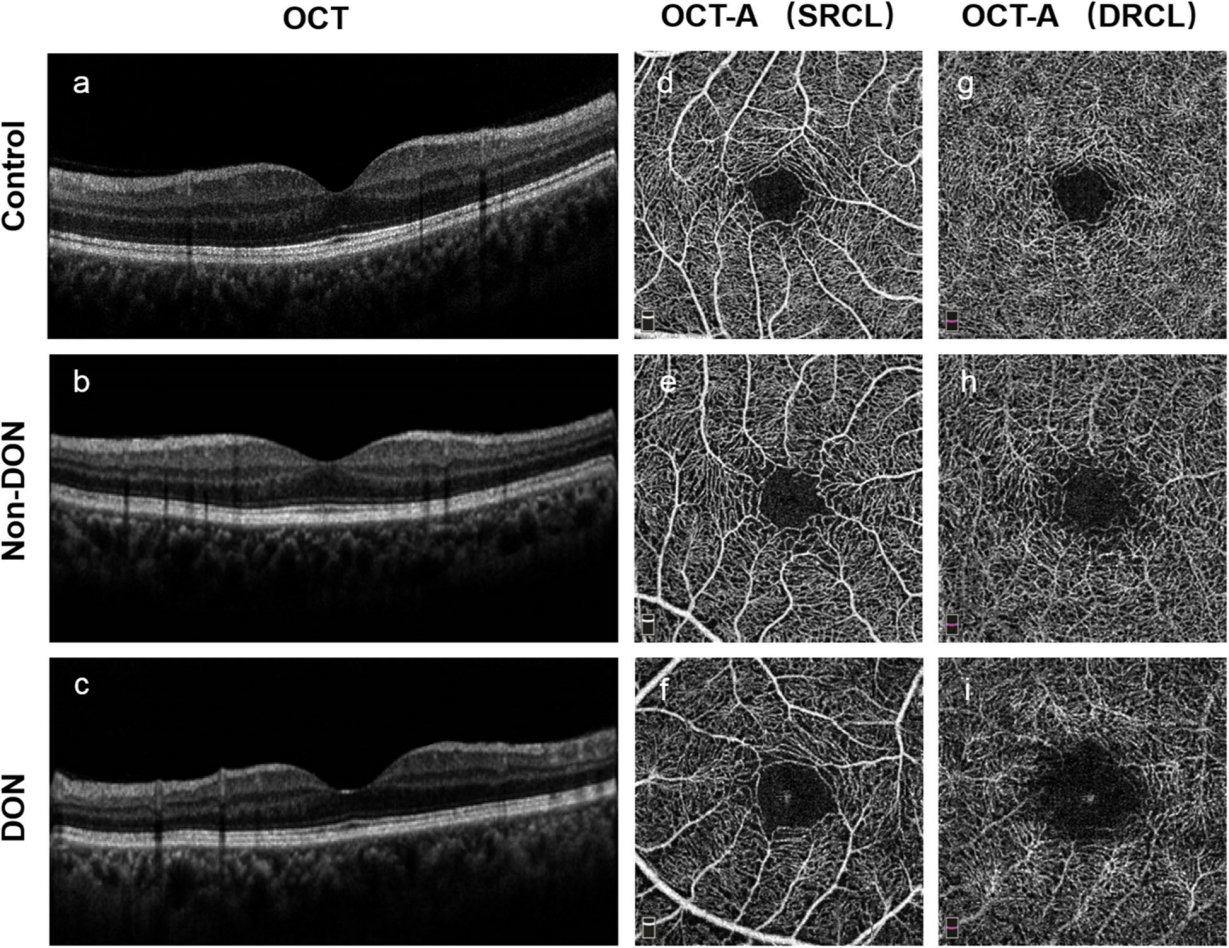
Representative OCT and OCT-A images of the superficial and deep capillary layers of a control eye (Control), an eye without DON, and an eye with DON. **a–c** OCT images in the horizontal scan of control, Non-DON, and DON subjects, respectively. **d-i** En face OCT-A images of the control, Non-DON, and DON subjects showing the SRCL (**d–f**) and DRCL (**g–i**). OCT, optical coherence tomography; OCT-A, optical coherence tomography angiography; DON, dysthyroid optic neuropathy; SRCL, superficial retinal capillary layer; DRCL, deep retinal capillary layer

**Fig. 2:**
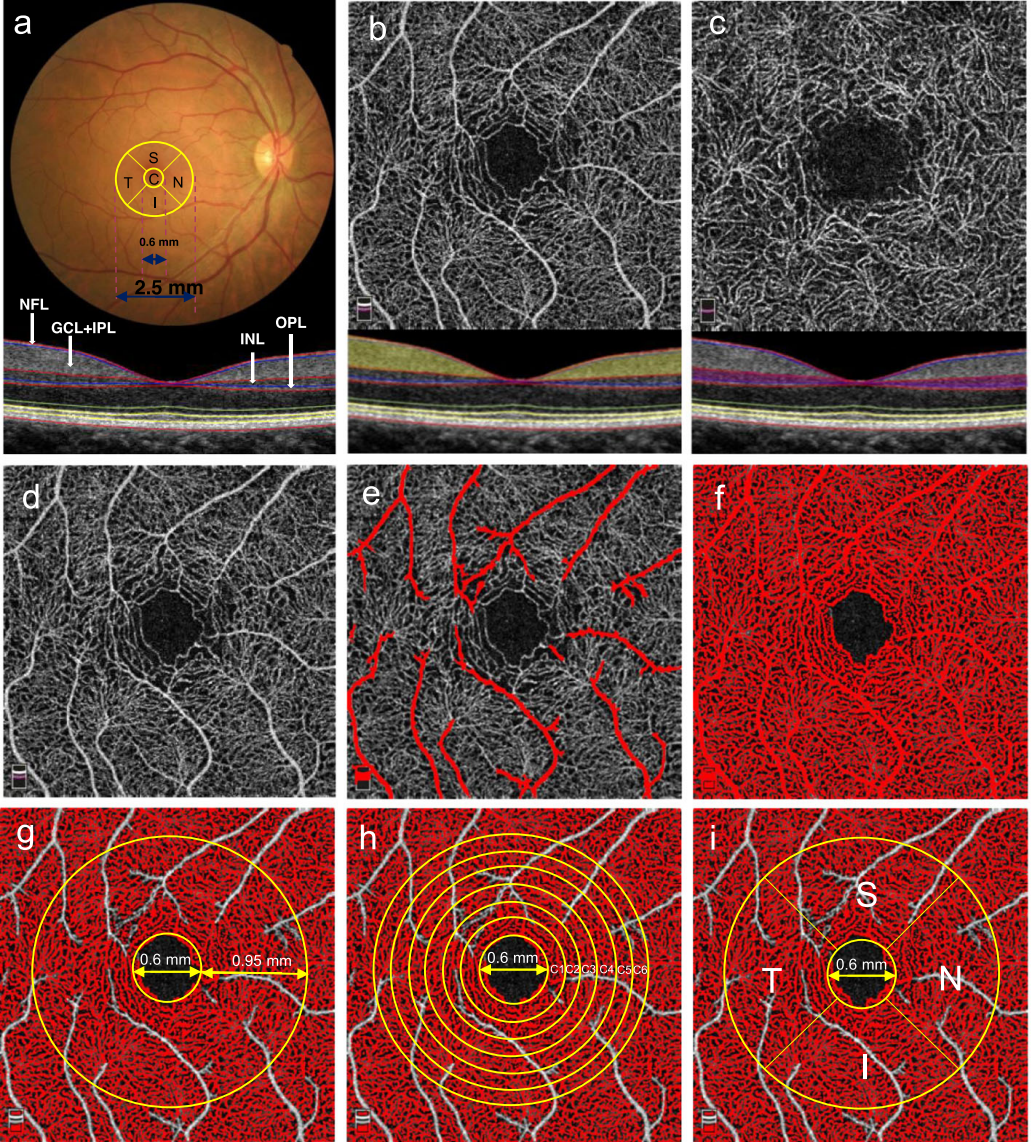
Imaging, segmentation, and postprocessing of the intra-retinal layers and the corresponding microvascular layers to determine the RCDs. (**a**, upper) The macula, 2.5 mm in diameter, included the central (C) region and four sectors: superior (S), temporal (T), inferior (I), and nasal (N). **a**, lower The four labeled intra-retinal layers in the SD-OCT image of the horizontal meridian included the NFL, GCL + IPL, INL, and OPL. **b**, lower NFL + GCL + IPL (yellow) corresponding to the (**b**, upper) SRCL. **c**, lower INL + OPL (purple) corresponding to the (**c**, upper) DRCL. **d** Original OCT-A image of the SRCL. **e** The automated algorithm identified only the large blood vessels in panel (**d**). **f** The automated algorithm identified both the large and small vessels. **g-i** The postprocessed images were obtained by subtracting panel (**e**) from panel (**f**). **g** The RCD was determined on the TAZ with a diameter of 2.50 mm after excluding the FAZ (diameter = 0.60 mm). **h** The RCD was determined in six annular zones, C1 (diameter = 0.92 mm), C2 (diameter = 1.23 mm), C3 (diameter = 1.55 mm), C4 (diameter = 1.87 mm), C5 (diameter = 2.18 mm), and C6 (diameter = 2.50 mm). **i** The RCD was determined in the parafoveal four quadrant sectors, S, T, I, and N, of a circular zone with a diameter of 2.50 mm after excluding the FAZ. RCD, retinal capillary density; SD-OCT, spectral domain optical coherence tomography; NFL, nerve fiber layer; GCL, ganglion cell layer; IPL, inner plexiform layer; INL, inner nuclear layer; OPL, outer plexiform layer; SRCL, superficial retinal capillary layer; DRCL, deep retinal capillary layer; OCT-A, optical coherence tomography; TAZ, total annular zone; FAZ, foveal avascular zone

All enrolled subjects were imaged by a commercial SD-OCT device (Optovue RTVue XR Avanti; Optovue, Inc., Fremont, CA, USA) that was equipped with Angiovue for OCT-A (Fig. 1). Radial scans (8 mm diameter; 18 lines) around the macula were performed to generate three-dimensional macular thickness maps (Fig. 2a). In addition, the OCT-A mode (3 × 3 mm area) was also used to obtain macular microvascular images (Fig. 2b and c) of the superficial and deep retinal capillary layers (SRCL and DRCL).

### Quantitative analysis of OCT images based on retinal thickness

As described previously [16, 23], the magnification of each cross-sectional retinal image was adjusted by the axial length and was then analyzed by one masked reader (YW) to segment the nine boundaries of the intra-retinal layers (Fig. 2a). The boundaries were derived through a dedicated software program based on gradient information and shortest path search protocol developed in MATLAB (The MathWorks Inc., Natick, MA, USA). The thickness of each intra-retinal layer was calculated by subtracting the boundary positions of each of the adjacent layers obtained by automated segmentation along the depth. In the current study, only the nerve fiber layer (NFL) between the first and second boundaries, the ganglion cell layer and the inner plexiform layer (GCL + IPL) between the second and third boundaries, and the ganglion cell complex (GCC), which included the NFL and GCL + IPL, were investigated (Fig. 2a).

To match the areas of retinal structure and microvasculature, a 2.5 mm diameter 3D thickness map of the NFL, GCL + IPL, and GCC in each eye was generated based on the segmented layers around the macula (Fig. 2a). For analysis, the macular thickness map was divided into a 0.6 mm diameter circle centered over the fovea and a total annular zone (TAZ) surrounded by a concentric ring 0.3 to 1.25 mm from the fovea. The areas of the central circle and TAZ were then divided into five regions including the central region (C) and the superior, temporal, inferior, and nasal sectors (S, T, I, and N, respectively). The mean thicknesses for the NFL, GCL + IPL, and GCC in each region and sector were calculated.

### Quantitative analysis of OCT-A images based on retinal capillary density

The OCT-A image size was 304 × 304 pixels and sets of high quality scans with signal strength indices greater than 40 were selected for further analysis. The SRCL extended from 3 μm below the internal limiting membrane to 15 μm below the inner plexiform layer (IPL), which supplies nutrients to the NFL and GCL + IPL (Fig. 2b). The DRCL, which supplies nutrition for the inner nuclear layer (INL) and outer plexiform layer (OPL), extended from 15 to 70 μm below the IPL (Fig. 2c).

Retinal capillary density (RCD, %) was defined as the proportion of the measured area occupied by flowing blood vessels, measured as pixels having decorrelation values acquired by the split-spectrum amplitude-decorrelation angiography algorithm [24, 25]. It was used to characterize the vascular structural information and was calculated by our custom automated algorithm based on the imported raw OCT-A images in PNG format [26]. To calculate the RCD, the grayscale of each two-dimensional OCT-A image was first extended by bicubic interpolation to 1024 × 1024 pixels to enhance the image details. Second, a two-way combined method consisting of a canny edge detector algorithm and a level set algorithm was used to detect the boundary of the foveal avascular zone. The area within the foveal avascular zone, composed of a 0.6 mm fixed diameter circle, was then determined to establish the baseline signal-to-noise ratio for global thresholding. Third, this image was then separately processed to generate two binary images by global thresholding and adaptive thresholding (Fig. 2d). The first image contained only the large blood vessels (Fig. 2e). The second binary image contained the large and small vessels (Fig. 2f). The two resulting binary vessel maps were subtracted to obtain the binary image containing only the small vessels (Fig. 2g-i). The RCD was then calculated for the 2.5 mm diameter TAZ (Fig. 2g) after excluding the foveal avascular zone (diameter = 0.6 mm), six concentric isometric annular rings (C1-C6) (Fig. 2h), and the S, T, I, and N parafoveal quadrant sectors (Fig. 2i) in the OCT-A images. All the above methods were implemented using MATLAB v.2015a (MathWorks, Inc.).

### Statistical methods

All data were expressed as means ± standard deviations and were analyzed with SPSS software (version 22.0; SPSS, Inc., Chicago, IL, USA). The generalized estimating was used to adjust the inter-correlation between two eyes from the same patient with respect to intra-retinal layer thickness and microvascular density. One-way analysis of variance was used to test for differences among the control subjects, non-DON, and DON groups. Post hoc tests were used for group pairs. Pearson’s correlation was used to analyze the relationships among the GCC thicknesses and the RCD of the SRCL. The receiver operating characteristic (ROC) curve was calculated to evaluate the ability of the OCT-based inner intra-retinal layer thicknesses and OCT-A-based RCD to determine the impairment of the retinal structure and microvasculature in the clinically diagnosed DON cases. Larger areas under the ROC curve (AUC) indicated higher diagnostic values. In addition, a prediction probability was calculated by the linear combination of the two indexes through the multivariate logistic regression model [27]. The AUC was then calculated by using the prediction probability. *P* <  0.05 was considered statistically significant in general comparisons. However, we set the significant *P* value to < 0.01 in multiple testing for the RCDs and thicknesses to improve the statistical validity.

## Results

### Patient characteristics

Forty-four consecutive patients with TAO [23 with non-DON (36 eyes); 21 with DON (38 eyes)] and 38 control subjects (38 eyes) were included. Except for the IOP and BCVA (*P* <  0.001, *P* = 0.005 respectively) there were no significant differences among the three groups in age, sex, spherical equivalent, and axial length (*P* = 0.174–0.979, Table 1). There were no significant differences of the exophthalmometry (*P* = 0.228), duration (*P* = 0.356), smoking (*P* = 0.192), FT3 (*P* = 0.192) and FT4 (*P* = 0.389) between the two TAO groups. However, the visual field MD and the CAS score of the DON group were higher than for the non-DON group (*P* <  0.001 and *P* = 0.019, respectively; Table 1).

**Table 1:** Demographic characteristics of all subjects

Parameters	Control (*n* = 38)	Non-DON (*n* = 23)	DON (*n* = 21)	*P* value
Eyes	38	36	38	–
Age (years)	45.2 ± 10.8	41.9 ± 11.7	47.6 ± 6.5	0.174*
Sex, Male/Female	19/19	12/11	11/10	0.979†
Spherical Equivalent (D)	−0.12 ± 0.95	−0.22 ± 0.66	−0.32 ± 0.63	0.521*
Intraocular Pressure (mmHg)	14.4 ± 2.3	16.1 ± 3.2	19.0 ± 6.9	< 0.001*
Axial Length (mm)	23.5 ± 0.9	23.3 ± 1.0	23.3 ± 0.8	0.520*
Best Corrected Visual Acuity (logMAR)	0.00 ± 0.01	0.00 ± 0.01	0.15 ± 0.37	0.005*
Exophthalmometry (mm)	–	17.94 ± 1.90	18.58 ± 2.38	0.228‡
Mean Defect of Visual Field (dB)	–	−0.29 ± 0.54	−6.79 ± 3.91	**< 0.001**‡
Clinical Activity Scoring	NA	1.08 ± 1.18	1.73 ± 1.12	**0.019**‡
Duration of Graves’ Disease (yrs)	NA	4.78 ± 7.03	3.20 ± 3.44	0.356‡
History of smoking	–	4/23	7/21	0.192†
Free Triiodothyronine (pg/ml)	–	7.13 ± 6.41	6.25 ± 4.76	0.192‡
Free Thyroxine (ng/dl)	–	3.41 ± 3.15	3.61 ± 5.51	0.389‡
Thyroid-Stimulating Hormone (μIU/ml)	–	7.21 ± 19.01	1.01 ± 1.37	**0.023**‡

Values for continuous variables are mean ± standard deviation for all subjects in each group. *Control =* control eyes; *Non-DON =* TAO patients without DON; *DON =* TAO patients with DON; *DON =* dysthyroid optic neuropathy; *TAO =* thyroid-associated ophthalmopathy; *– =* not performed; *NA =* not applicable. Bold *P*-value represents < 0.05

* ANOVA. † x^2^ test. ‡ t-test

### NFL, GCL + IPL, and GCC thickness

The thickness of the NFL, GCL + IPL, and GCC in both non-DON and DON groups were significantly decreased in almost all regions compared to the controls (*P* <  0.01, Table 2). However, there were no significant differences in the thickness of the NFL, GCL + IPL, or GCC between the two TAO groups (*P* = 0.039–0.958, Table 2). In addition, Table 2 also showed that the total retinal thicknesses of the T sector (*P* = 0.003) in non-DON group and the S sector (*P* = 0.004) in the DON group were significantly decreased compared with controls. However, the thickness of INL + outer retinal layer was not decreased either in the non-DON or DON groups (*P* > 0.01).

**Table 2:** Comparisons of inner intra-retinal layer thickness among controls and TAO patients with and without DON

Layers	Regions	Control (C)	Non-DON (N)	DON (D)	*P* Value (C vs. N)	*P* Value (C vs. D)	*P* Value (N vs. D)
NFL (μm)	C	6.9 ± 1.3	6.2 ± 1.7	6.9 ± 1.3	0.032	0.927	0.039
	TAZ	19.9 ± 3.2	17.7 ± 2.1	17.4 ± 1.3	**< 0.001**	**< 0.001**	0.585
	S	21.4 ± 3.6	19.1 ± 3.3	18.0 ± 1.9	**0.001**	**< 0.001**	0.126
	T	17.0 ± 2.7	15.5 ± 1.9	15.3 ± 1.9	**0.005**	**0.001**	0.681
	I	22.2 ± 3.6	19.1 ± 3.3	18.0 ± 1.9	**0.001**	**< 0.001**	0.920
	N	19.3 ± 4.6	16.3 ± 2.2	16.5 ± 1.8	**< 0.001**	**< 0.001**	0.778
GCL + IPL (μm)	C	14.6 ± 4.2	12.0 ± 4.1	12.0 ± 3.4	**0.005**	**0.005**	0.958
	TAZ	75.4 ± 8.3	69.6 ± 10.2	67.1 ± 13.1	0.021	**0.001**	0.347
	S	79.5 ± 9.6	71.6 ± 11.0	69.2 ± 14.2	**0.005**	**< 0.001**	0.387
	T	72.0 ± 13.4	65.1 ± 10.4	62.7 ± 12.8	0.018	**0.001**	0.406
	I	79.5 ± 9.6	71.6 ± 11.0	69.2 ± 14.2	**0.005**	**< 0.001**	0.337
	N	74.9 ± 9.8	66.8 ± 10.6	64.9 ± 13.2	**0.003**	**< 0.001**	0.479
GCC (μm)	C	21.5 ± 5.0	18.2 ± 5.0	18.9 ± 3.7	**0.002**	**0.015**	0.499
	TAZ	95.2 ± 10.3	87.3 ± 11.8	84.5 ± 13.4	**0.005**	**< 0.001**	0.341
	S	100.8 ± 12.0	90.6 ± 13.1	87.2 ± 15.0	**0.001**	**< 0.001**	0.270
	T	88.9 ± 14.5	80.6 ± 11.8	78.0 ± 11.9	**0.006**	**< 0.001**	0.386
	I	101.6 ± 13.1	92.7 ± 12.5	90.1 ± 14.0	**0.005**	**< 0.001**	0.400
	N	94.2 ± 13.2	83.1 ± 12.2	81.4 ± 13.2	**< 0.001**	**< 0.001**	0.581
INL + ORL (μm)	C	202.2 ± 41.2	202.9 ± 9.6	206.3 ± 26.4	0.921	0.549	0.623
	TAZ	219.3 ± 9.4	218.4 ± 7.1	221.4 ± 9.4	0.655	0.304	0.146
	S	219.6 ± 9.9	219.3 ± 7.5	221.6 ± 9.2	0.866	0.349	0.275
	T	221.8 ± 10.5	220.2 ± 8.2	224.0 ± 10.2	0.468	0.343	0.098
	I	216.5 ± 8.0	216.6 ± 7.2	219.9 ± 9.3	0.968	0.081	0.092
	N	218.6 ± 9.6	217.1 ± 6.8	220.5 ± 10.0	0.466	0.370	0.108
Total (μm)	C	213.3 ± 51.3	214.7 ± 14.9	215.8 ± 36.9	0.870	0.771	0.901
	TAZ	314.1 ± 17.5	305.4 ± 15.8	305.7 ± 12.8	0.019	0.021	0.937
	S	320.2 ± 19.5	309.7 ± 17.1	308.5 ± 14.8	0.011	**0.004**	0.772
	T	315.9 ± 21.8	302.9 ± 17.5	305.0 ± 14.9	**0.003**	0.012	0.635
	I	318.1 ± 19.1	309.2 ± 15.4	309.6 ± 12.9	0.020	0.025	0.915
	N	307.0 ± 21.5	297.1 ± 15.0	298.0 ± 11.5	0.012	0.021	0.806

*TAO =* thyroid-associated ophthalmopathy; *DON* = dysthyroid optic neuropathy; *Control (C)* = control subjects; *Non-DON (N) =* TAO patients without DON; *DON (D) =* TAO patients with DON; *NFL* = nerve fiber layer; *GCL + IPL =* ganglion cell layer and inner plexiform layer; *GCC* = ganglion cell complex; *INL =* inner nuclear layer; *ORL =* outer retinal layer; *C =* central region; *TAZ =* total annular zone; *S* = superior sector; *T =* temporal sector; *I =* inferior sector; *N =* nasal sector. Bold *P*-value represents < 0.01

### RCDs of the macular microvasculature

The RCDs in the SRCL for almost all regions were significantly lower in both the non-DON and DON groups compared to the controls (*P* <  0.01), except for the C2 region of both groups (*P* = 0.069 and 0.092, respectively), and C1 (*P* = 0.033) and C3 (*P* = 0.011) of the DON group. However, there were no significant differences of RCD in the SRCL between the two TAO groups (*P* = 0.127–0.899, Table 3). For the DRCL, the RCDs of the non-DON and DON groups were significantly lower in almost all regions compared to the controls (*P* <  0.01, Table 3). The exception was for the C1-C4 regions (*P* = 0.016–0.096) as well as T (*P* = 0.011) and N (*P* = 0.017) sectors of the non-DON group. In addition, the RCDs of the DON group in TAZ (*P* = 0.006), C4 (*P* = 0.004), C6 (*P* = 0.004), as well as T (*P* = 0.007), I (*P* = 0.005) and N (*P* = 0.007) sectors were lower than in the non-DON group (Table 3).

**Table 3:** Comparisons of the RCDs in the superficial and deep retinal capillary layers among controls and TAO patients with and without DON

Layers	Regions	Control (C)	Non-DON (N)	DON (D)	*P* Value (C vs. N)	*P* Value (C vs. D)	*P* Value (N vs. D)
SRCL (%)	TAZ	63.7 ± 3.3	60.0 ± 3.5	59.8 ± 4.5	**< 0.001**	**< 0.001**	0.807
	C1	57.6 ± 12.9	48.5 ± 14.6	51.1 ± 11.6	**0.003**	0.033	0.382
	C2	63.0 ± 5.6	60.6 ± 5.8	60.8 ± 5.2	0.069	0.092	0.869
	C3	64.0 ± 4.5	61.0 ± 4.3	61.2 ± 5.5	**0.008**	0.011	0.867
	C4	64.4 ± 3.7	61.4 ± 4.0	60.3 ± 5.3	**0.003**	**< 0.001**	0.326
	C5	64.8 ± 3.1	60.8 ± 3.8	60.9 ± 5.0	**< 0.001**	**< 0.001**	0.899
	C6	64.2 ± 2.9	61.4 ± 3.6	60.0 ± 4.4	**0.001**	**< 0.001**	0.127
	S	63.4 ± 3.9	60.3 ± 4.3	60.0 ± 5.3	**0.004**	**0.001**	0.789
	T	64.0 ± 3.5	61.2 ± 4.2	60.3 ± 5.0	**0.005**	**< 0.001**	0.385
	I	63.7 ± 3.6	59.7 ± 4.0	59.5 ± 5.6	**< 0.001**	**< 0.001**	0.857
	N	63.6 ± 4.3	59.0 ± 4.0	59.5 ± 4.5	**< 0.001**	**< 0.001**	0.652
DRCL (%)	TAZ	73.9 ± 4.8	70.2 ± 4.4	66.4 ± 7.7	**0.007**	**< 0.001**	**0.006**
	C1	40.6 ± 18.8	34.0 ± 16.2	29.7 ± 15.3	0.096	**0.006**	0.277
	C2	71.6 ± 8.5	66.7 ± 8.4	61.3 ± 12.4	0.039	**< 0.001**	0.020
	C3	78.3 ± 4.1	75.2 ± 4.3	71.9 ± 7.5	0.016	**< 0.001**	0.013
	C4	78.4 ± 4.0	75.9 ± 3.6	72.3 ± 7.4	0.045	**< 0.001**	**0.004**
	C5	77.8 ± 3.6	74.4 ± 3.9	71.3 ± 7.7	**0.007**	**< 0.001**	0.017
	C6	76.5 ± 4.2	72.9 ± 4.1	68.9 ± 8.1	**0.009**	**< 0.001**	**0.004**
	S	75.0 ± 4.6	71.2 ± 4.1	68.0 ± 8.1	**0.008**	**< 0.001**	0.020
	T	73.4 ± 4.7	69.4 ± 5.1	65.1 ± 9.2	0.011	**< 0.001**	**0.007**
	I	75.0 ± 4.9	71.5 ± 4.9	67.7 ± 7.0	**0.009**	**< 0.001**	**0.005**
	N	72.4 ± 5.7	68.8 ± 4.6	64.8 ± 7.9	0.017	**< 0.001**	**0.007**

*RCD* = retinal capillary density; *TAO =* thyroid-associated ophthalmopathy; *DON =* dysthyroid optic neuropathy; *Control (C) =* control subjects; *Non-DON (N) =* TAO patients without DON; *DON (D) =* TAO patients with DON; *SRCL =* superficial retinal capillary layer; *DRCL =* deep retinal capillary layer; *C =* central region; *TAZ* = total annular zone; *C1~C6* = six annular zones; *S =* superior sector; *T =* temporal sector; *I =* inferior sector; *N =* nasal sector. Bold *P*-value represents < 0.01

### Relationships among the inner retinal layer thickness, the macular microvasculature RCD and VA

**Fig. 3:**
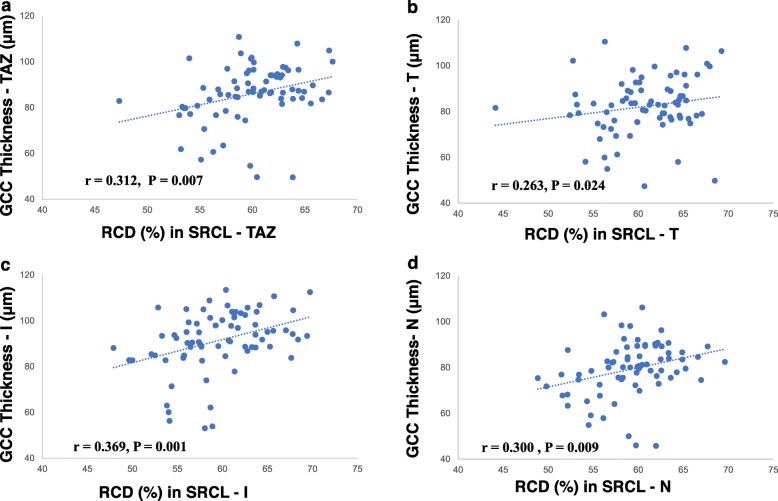
Correlations between GCC thickness and microvascular density in TAO patients. **a** Correlation between the GCC (including NFL + GCL + IPL) thickness and SRCL RCD in the TAZ region (*r* = 0.312, *P* = 0.007). **b** Correlation between GCC thickness and SRCL RCD in the T sector (*r* = 0.263, *P* = 0.024). **c** Correlation between GCC thickness and SRCL RCD in the I sector (*r* = 0.369, *P* = 0.001). **d** Correlation between GCC thickness and SRCL RCD in the N sector (*r* = 0.300, *P* = 0.009). TAO, thyroid-associated ophthalmopathy; GCC, ganglion cell complex; NFL, nerve fiber layer; GCL, ganglion cell layer; IPL, inner plexiform layer; RCD, retinal capillary density; SRCL, superficial retinal capillary layer; TAZ, total annular zone; T, temporal sector; I, inferior sector; N, nasal sector

The GCC thickness in both TAO groups was positively correlated with the RCD of the SRCL in the TAZ (*r* = 0.312, *P* = 0.007, Fig. 3a) and in the T (*r* = 0.263, *P* = 0.024, Fig. 3b), I (*r* = 0.369, *P* = 0.001, Fig. 3c), and N sectors (*r* = 0.300, *P* = 0.009, Fig. 3d). We also found that the NFL thickness (*r* = − 0.279, *P* = 0.030) and the RCDs (C6 region: *r* = − 0.286, *P* = 0.020; T sector: *r* = − 0.282, *P* = 0.030) in the SRCL were negatively correlated with the BCVA.

### ROC curve analysis

**Fig. 4:**
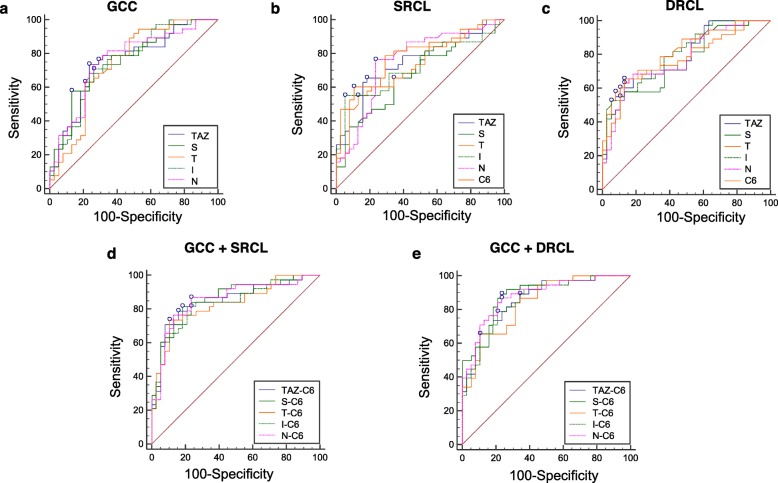
ROC analysis of the GCC thickness and the microvascular density in the TAO patients with DON. **a** AUCs of the GCC thickness in the TAZ and the S, T, I, and N sectors. **b** AUCs of SRCL RCD in the TAZ, the C6 annular zone, and the S, T, I, and N sectors. **c** AUCs of the DRCL RCD in the TAZ, the C6 annular zone, and the S, T, I, and N sectors. **d** AUCs of the combined indicators CGG thickness in the TAZ and the S, T, I, and N sectors along with the SRCL RCD in the C6 annular zone. **e** AUCs of the combined indicators CGG thickness in the TAZ and the S, T, I, and N sectors along with the DRCL RCD in the C6 annular zone. ROC, receiver operating characteristic; RCD, retinal capillary density; GCC, ganglion cell complex; TAO, thyroid-associated ophthalmopathy; DON; dysthyroid optic neuropathy; AUC; areas under the ROC curve; TAZ, total annular zone; S, superior sector; T, temporal sector; I, inferior sector; N, nasal sector; SRCL, superficial retinal capillary layer; DRCL, deep retinal capillary layer

We performed ROC curve analysis to determine the ability of the retinal thickness and microvascular density to distinguish the TAO patients with DON from the controls. The AUCs of the GCC thickness ranged from 0.74 to 0.76 (Fig. 4a). In addition, the AUCs of the RCD in the SRCL and DRCL ranged from 0.64 to 0.79 (Fig. 4b), and from 0.66 to 0.81 (Fig. 4c), respectively. We combined the indicators of GCC thickness in the TAZ and in the S, T, I, and N sectors with the RCD of the SRCL and the DRCL in the C6 region, which had the largest AUC. We found that the AUCs of the composite index were generally larger than those of each single indicator, with the largest in the S-C6. For the SRCL, the AUC = 0.86, sensitivity = 82%, and specificity = 82% (*P* <  0.001, Fig. 4d, Table 4). For the DRCL, the AUC = 0.89, sensitivity = 89%, and specificity = 76% (*P* <  0.001, Fig. 4e, Table 4). The smallest was in the T-C6, where for the SRCL, the AUC = 0.83, sensitivity = 74%, specificity = 89% (*P* <  0.001, Fig. 4d, Table 4). For the DRCL, the AUC = 0.84, sensitivity = 66%, specificity = 89% (*P* <  0.001, Fig. 4e, Table 4).

**Table 4:** ROC Curve Analysis of RCDs in the Retinal Capillary Layers and GCC Thickness in TAO Patients with DON

	GCC + SRCL					GCC + DRCL				
		Cut off					Cut off			
Regions	AUC	GCC (μm)	RCD (%)	Sensitivity (%)	Specificity (%)	AUC	GCC (μm)	RCD (%)	Sensitivity (%)	Specificity (%)
TAZ-C6 Total	0.85	83.2	64.6	79	84	0.86	83.2	79.5	89	66
S-C6	0.86	79.3	66.9	82	82	0.89	86.2	79.5	89	76
T-C6	0.83	92.8	60.2	74	89	0.84	95.4	68.6	66	89
I-C6	0.83	107.8	61.0	82	76	0.86	124.8	66.4	79	79
N-C6	0.85	95.7	61.0	87	76	0.88	80.7	78.6	87	76

*RCD* = retinal capillary density; *GCC =* ganglion cell complex; *TAO =* thyroid-associated ophthalmopathy; *DON =* dysthyroid optic neuropathy; *SRCL =* superficial retinal capillary layer; *DRCL =* deep retinal capillary layer; *AUC =* areas under the ROC curve; *TAZ =* total annular zone; *S =* superior sector; *T =* temporal sector; *I =* inferior sector; *N =* nasal sector

## Discussion

TAO is an autoimmune inflammation of the orbital tissues and is characterized by enlarged extraocular muscles and increased orbital fat. It is associated with orbital fibroblasts, immune cells, cytokines, autoantibodies, genetics, and environmental factors [28, 29]. Increased intraorbital pressure and the swelling of the extraocular muscles cause compressive optic neuropathy, retinopathy, and visual function loss. To provide morphological evidence relevant to the pathogenic mechanisms of DON, we imaged in vivo the inner intra-retinal layers and quantified the layer thicknesses and the microvascular density around the macula in both non-DON and DON patients. In addition, we analyzed the ability of these morphological indicators to detect the early retinal changes of these patients.

We found that the inner intra-retinal layers around the macula, including the NFL, GCL + IPL, as well as the GCC, were significantly decreased in the TAO patients, with or without DON. Sen et al. [11] and Blum Meirovitch et al. [12] reported that the total macular thickness was significantly thinner in TAO patients, and they speculated that this might be caused by thinning of the GCC. In our study, we used a high resolution spectral domain OCT instrument that readily imaged the eight intra-retinal layers from the NFL to the retinal pigment epithelium [30]. We found that, in support of the speculation by Meirovitch et al. [12], the thicknesses of the NFL, GCL + IPL, and GCC were all decreased in both TAO subgroups and are likely responsible for the thinning of the whole retina around the macula. Zhang et al. [31] also reported similar decreases of the GCC layer thickness in the DON group. Mechanical compression of the retina by orbital contents, decreased blood supply, as well as secondary increases in IOP were considered as possible reasons for ganglion cell atrophy resulting in macular thinning in the eyes with TAO [12]. In addition, because TAO has an immunologic component, the presence of auto-anti-retinal antibodies might play a role in retinal thinning similar to patients with autoimmune retinopathy [32].

In other studies, the P-RNFL thickness was reported to be significantly thinner in TAO patients [10, 11, 13], which is consistent with the macular NFL thinning in our study. However, Meirovitch et al. [12] found that the P-RNFL thickness did not decrease, but rather became thicker compared with normal eyes. They considered that the disk edema derived from the orbital inflammation might be the reason for the thickening. In this study, we found that the inner retinal thickness was thinner in both TAO groups compared to the controls, and there was no difference between the non-DON and DON groups. We speculate that ganglion cell atrophy and inflammatory edema coexist in DON patients of the current study. Most were still at the early stage of DON (MD of visual field: − 6.79 ± 3.91 dB) and had higher CAS values than did the non-DON group, reflecting higher clinical activity [33]. We will conduct further studies to investigate the intra-retinal structure in DON patients at a more advanced disease stage.

The microvasculature around the macula supplies oxygen and nutrition to the retina and is vital for supporting metabolism in the macular tissue. Sayin et al. [13] found that in TAO patients, the temporal and inferior P-RNFL thickness of the left eye were significantly thinner than in the right eye even though there were no significant differences in proptosis or IOP between the eyes. They speculated that asymmetric blood flow and lymphatic drainage resulting from orbital structural differences might be responsible in part for these findings [34, 35]. In the current study, we found that the RCDs in both the SRCL and DRCL of the two TAO groups were lower than in the controls. Similar results were also reported by Zhang et al. [31] though they only detected the vascular density in the SRCL. In addition, we found that the RCDs in SRCL and the GCC thickness were positively correlated in the TAZ region and in the T, I, and N sectors. This indicated that microvasculature loss and inner retinal thickness loss were co-existent, however, it might still be impossible to distinguish the causal effect between them. Furthermore, the RCDs in the DON group were significantly lower than in the non-DON group. The higher proptosis and IOP values in the DON patients enrolled in our study might have contributed to these findings. In addition, Chu et al. [36] found that the plasma level of ET-1, a strong vasoconstrictor, was higher than normal in thyroid hormone disorders caused by Graves’ disease. ET-1 can decrease blood flow, which might be another reason for the lower RCDs around the macula. We speculate that the continually decreasing blood supply might be responsible for the transition from non-DON to DON. However, Ye et al. [37] reported that the macular microvascular densities were significantly increased in active TAO patients. This discrepancy might be ascribed to the difference in the study design in which Ye et al.’s subjects were primarily active TAO patients uncomplicated with DON.

Most previous studies focused on TAO patients or only DON patients. Seldom has a study been performed to record the characteristics of TAO patients without DON and who appear normal with respect to visual function, optic nerve head appearance, and CT scan results [31]. We found that both the inner intra-retinal layer thickness and the RCDs in SRCL and DRCL were significantly decreased in almost all regions and sectors compared to the controls. This finding suggests that the morphological changes of the fundus occur prior to clinical changes in visual function. Thus, ophthalmologists should pay more attention to the indicators of retinal structure and microvasculature around the macula of TAO patients. Observations made there might be valuable in the early detection, intervention, and monitoring for DON.

To determine the ability of the morphological indicators around the macula to detect impairment of the retinal structure and microvasculature in TAO patients with mild to moderate DON, we performed ROC curve analyses. We found that the AUCs for GCC thickness and the RCDs in the SRCL and the DRCL separately had relatively little ability to discriminate between DON patients and controls. However, interestingly, the AUCs became larger after combining the GCC thickness and the RCDs in the SRCL or DRCL. This implies that there is a close connection between the retinal structure and microvasculature in these patients. Therefore, ophthalmologists should undertake a comprehensive consideration of the retinal structure and microvasculature in estimating and treating the early retinopathy or optic neuropathy of TAO patients.

There were some limitations in the current study. First, we only analyzed a limited area in the parafoveal region around the macula i.e., a 2.5 mm diameter around the fovea. It is likely that significant information in larger fields around the macula was present but not detected. Second, we did not investigate the characteristics of the P-RNFL and the microvasculature around the optic nerve head. These data will certainly be valuable either in diagnosing or monitoring DON, especially when combined with the indicators from around the macula. Future studies will explore these possibilities. Third, the laboratory serum biochemical indicators like FT3, FT4 and TSH as well as the visual field and exophthalmometry of the control participants were not examined in the study. However, these data may not impact the results because it would most likely have led to an underestimate of the difference in retinal thickness and microvascular density between the TAO patients and the controls instead of an overestimate. Fourth, the retinal thickness and microvascular density in the TAO patients might have been affected by several risk factors, like proptosis, IOP, activity, as well as the thyroid-related laboratory serum biochemical indicators, which will be explored in our future study. Last, most of the patients in the DON group had relatively mild signs and were in the early stage of DON. We will report in more depth on the DON group in future longitudinal studies with larger sample sizes to monitor the development of the retinal structural and the microvascular damage during the transition from mild to severe conditions, as well as the differences between the active and quiescent stages.

## Conclusions

In summary, in TAO patients with or without DON, the inner intra-retinal layers were thinner, and the density of the microvascular network was decreased compared to control eyes. The current study also suggests that the morphological changes in retinal structure and the surrounding microvasculature might precede visual function deficits detected in clinical tests. The combination of retinal structure and microvascular density indicators might be valuable in the diagnosis, monitoring, and intervention in the early stages of DON onset, which should be further verified by longitudinal studies with larger sample sizes. Spectral domain OCT and OCT-A technologies offer new paths to study TAO, and the diagnostic indicators will likely be useful in the future as objective biomarkers for predicting which patients are likely to transition from non-DON to DON.

## Data Availability

All relevant data are in the manuscript together with its supporting files.
